# Double-balloon enteroscopy for retrieval of dental prosthesis partially embedded in the jejunum for over 2 months

**DOI:** 10.1016/j.igie.2023.04.001

**Published:** 2023-05-11

**Authors:** Yi Lu, Di Zhang, Wenru Li, Dan Huang, Jiachen Sun

**Affiliations:** 1 Department of Gastrointestinal Endoscopy; 2 Guangdong Provincial Key Laboratory of Colorectal and Pelvic Floor Diseases; 3 Department of Anorectal Surgery; 4 Department of Radiology, The Sixth Affiliated Hospital, Sun Yat-sen University, Guangzhou, People's Republic of China; 5 Department of Gastroenterology, The People's Hospital of Guangxi Zhuang Autonomous Region, Nanning, People's Republic of China

A 60-year-old woman was admitted to our department after swallowing her dental prosthesis over 2 months earlier. Because she did not have any discomfort and did not know the harm of ingesting a dental prosthesis, she did not tell her daughter, until her daughter realized the dental prosthesis was missing.

Palpation revealed no pathologic findings, and laboratory values for blood, stool, and C-reactive protein were normal. An abdominal radiograph showed a high-density shadow in the left lower abdomen (Fig. 1), and an abdominal CT identified the dental prosthesis in the jejunum (Fig. 2). Double-balloon enteroscopy (DBE) was used to remove the prosthesis (Video 1, available online at www.igiejournal.org).

**Figure 1:**
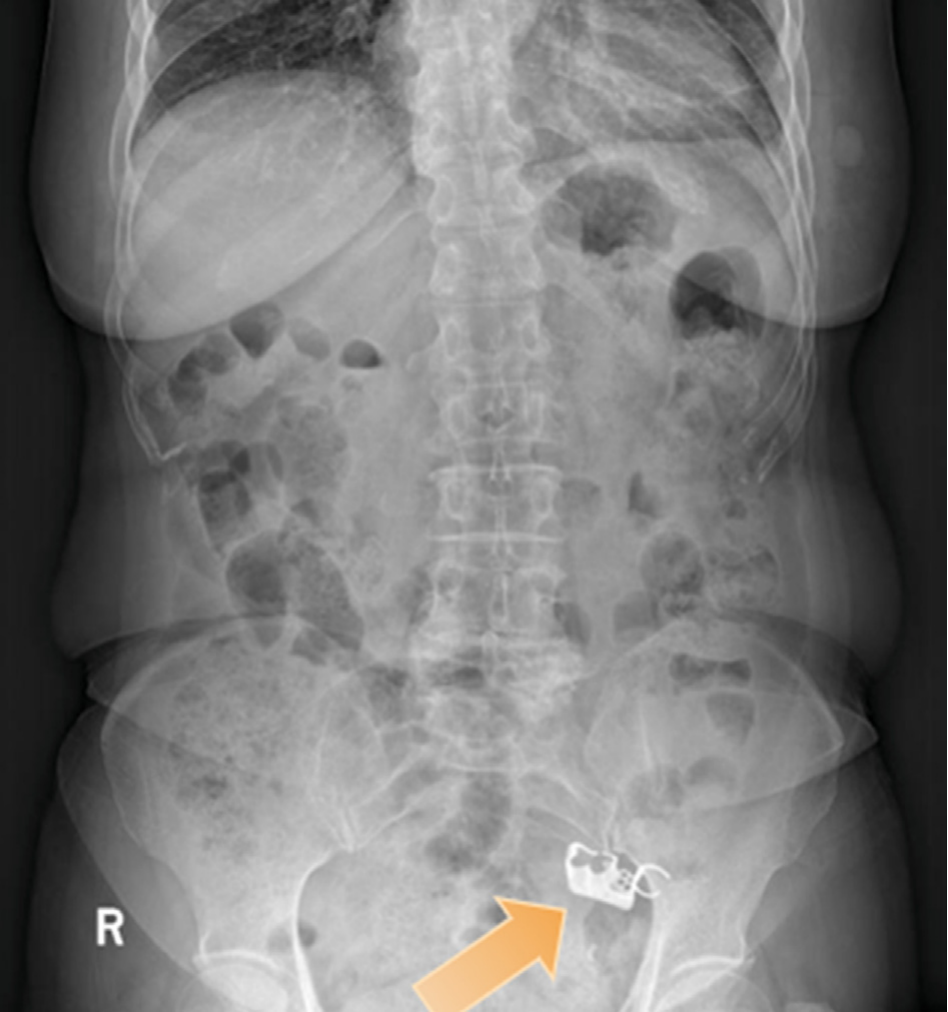
Abdominal radiograph showed a high-density shadow in the left lower abdomen.

**Figure 2:**
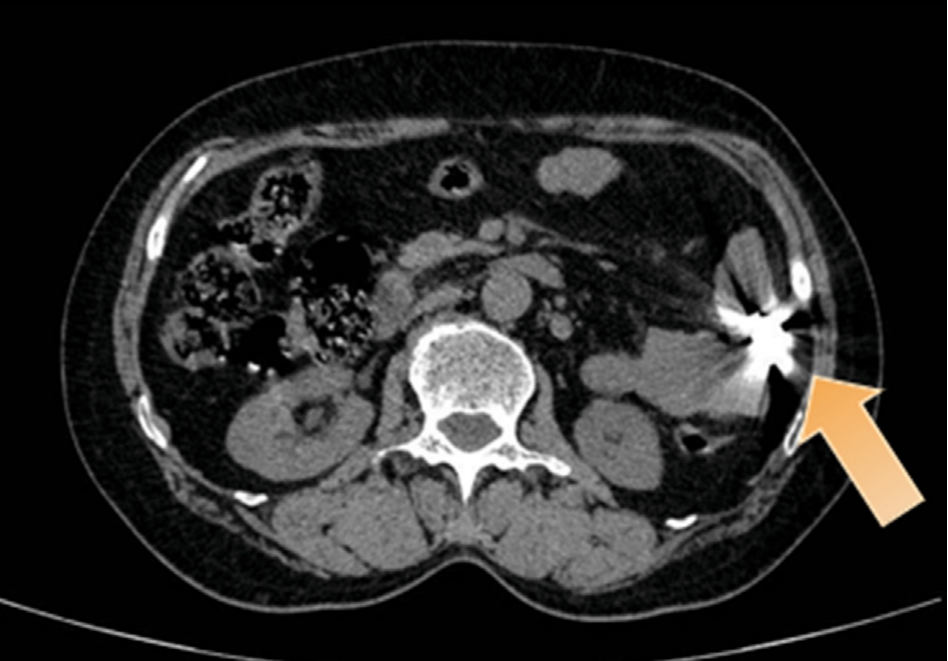
Abdominal CT identified the dental prosthesis in the jejunum.

Because at our center DBE is used without a cap, we made a cap to avoid injury to the small intestine (Fig. 3). We took a cap used for gastroduodenoscopy and used adhesive tape to make it smaller to fit the enteroscope. We found the dental prosthesis partially embedded and ulcerated in the jejunum about 60 cm from the pylorus (Fig. 4). The embedded sharp part (the hook) was in the intestinal wall, and we dragged the prosthesis with a snare, attempting to hide the sharp part of the prosthesis in the cap to avoid mucosal injury. However, because it was difficult to drag the prosthesis and it caught at the turning point, we changed the direction of the snare to catch the hook and then the prosthesis parallel to the intestine and were able to successfully retrieve it without causing any other injury to the stomach or esophagus (Fig. 5). Finally, we reinspected the site after retrieval of the dental prosthesis and used a clip to close the ulceration and small injury in the jejunum. No adverse events occurred after the endoscopic treatment, and the patient recovered without any discomfort.

**Figure 3:**
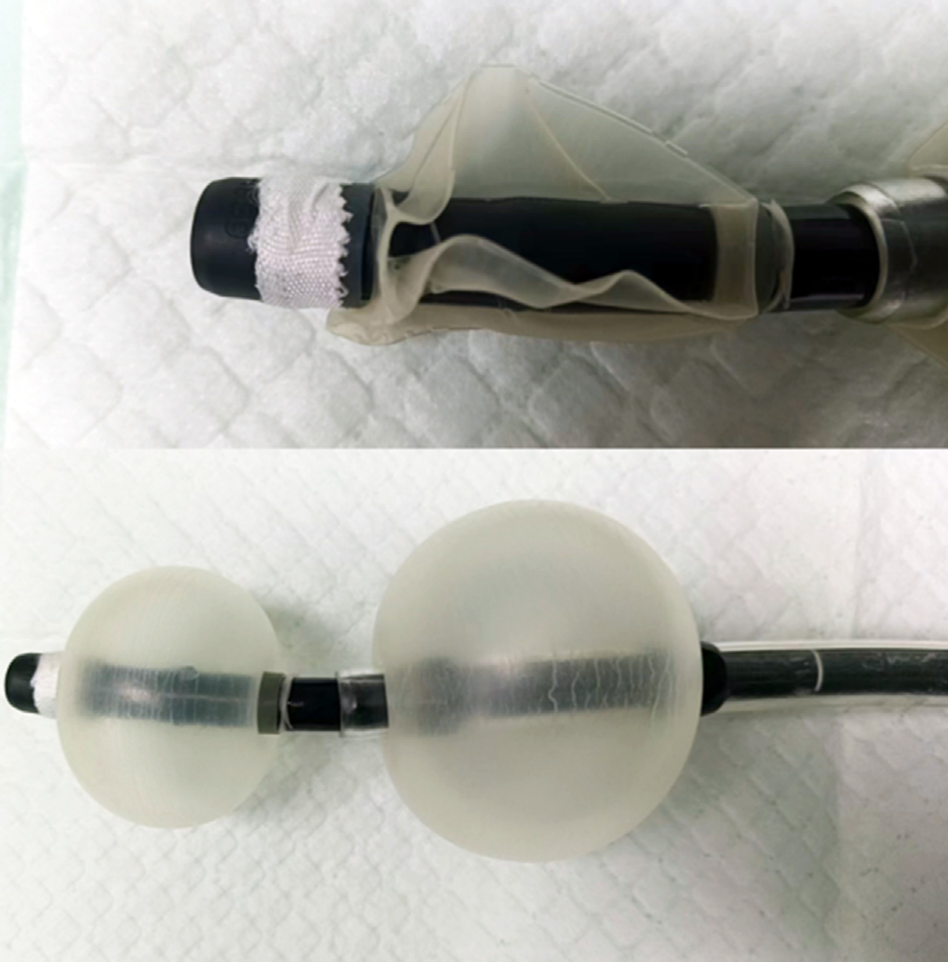
The cap for the double-balloon enteroscopy we made to avoid injury to the small intestine.

**Figure 4:**
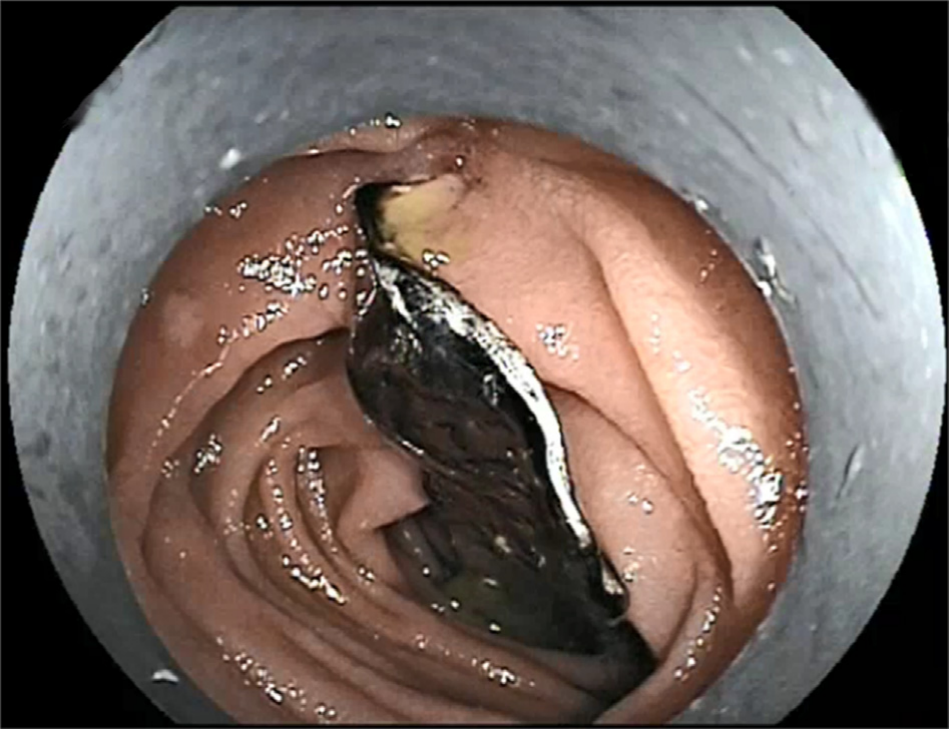
The dental prosthesis was located in the jejunum, partially embedded and with ulceration.

**Figure 5:**
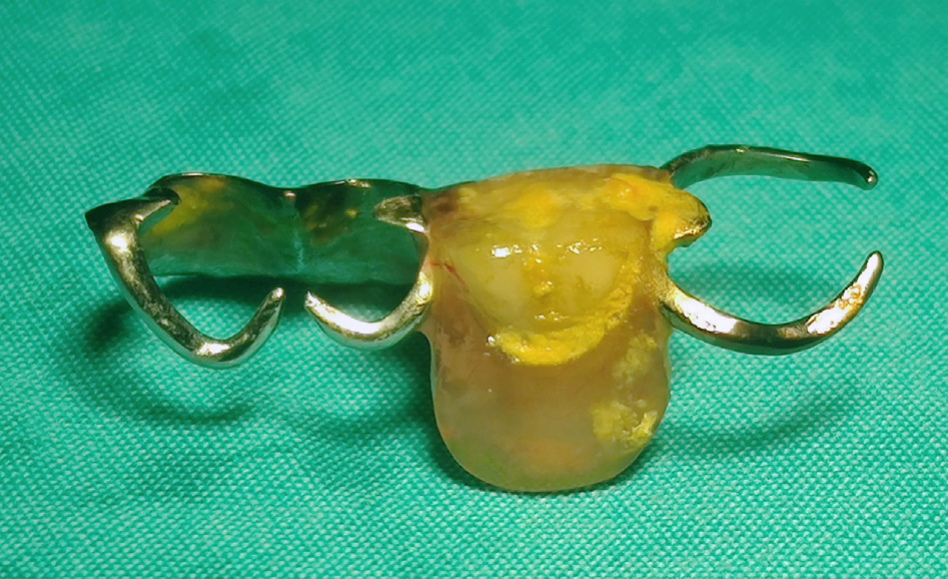
The removed dental prosthesis.

Normally, foreign objects making it asymptomatically into the small bowel beyond the ligament of Treitz are left alone unless they become symptomatic. In this case, however, although the patient was asymptomatic, the foreign body was a dental prosthesis, which is very sharp, harboring the risk for intestinal injury and even perforation. For this patient, the dental prosthesis had been embedded in the jejunum for over 2 months, and we believed a watch-and-wait strategy was not the best policy and retrieval should be attempted as soon as possible. The deep ulcers in the small intestine proved that our decision was correct. In this case, we used a soft cap created especially for the enteroscope to retrieve the sharp part of the prosthesis into the cap to protect the intestine from injury and without influencing the balloon’s inflation. The dental prosthesis was successfully retrieved, avoiding the need for surgery. This indicates the usefulness of DBE for nonsurgical retrieval of impacted foreign bodies in the small intestine.^1^

## Patient Consent

The authors have received appropriate patient consent for the publication of this article.

## Disclosure

*All authors disclosed no financial relationships.*

## Appendix

Video 1 The dental prosthesis was located in the jejunum, partially embedded and with ulceration, and was removed successfully by double-balloon enteroscopy with a snare.
